# Identification of tumor mutations in plasma based on mutation variant frequency change (MVFC)

**DOI:** 10.1002/1878-0261.13498

**Published:** 2023-08-07

**Authors:** Geng Chen, Fang Peng, Xiuqing Dong, Zhixiong Cai, Zhenli Li, Lei He, Jinpan Hu, Xiaoxu Deng, Yutong Guo, Liman Qiu, Yang Zhou, Jingfeng Liu, Huqin Zhang, Xiaolong Liu

**Affiliations:** ^1^ School of Life Science and Technology Xi'an Jiaotong University China; ^2^ The United Innovation of Mengchao Hepatobiliary Technology Key Laboratory of Fujian Province Mengchao Hepatobiliary Hospital of Fujian Medical University Fuzhou China; ^3^ Mengchao Med‐X Center Fuzhou University China

**Keywords:** cfDNA, hepatocellular carcinoma, liquid biopsy, minimum residual disease, variant frequency change

## Abstract

To overcome the dependency of strategies utilizing cell‐free DNA (cfDNA) on tissue sampling, the emergence of sequencing panels for non‐invasive mutation screening was promoted. However, cfDNA sequencing with panels still suffers from either inaccuracy or omission, and novel approaches for accurately screening tumor mutations solely based on plasma without gene panel restriction are urgently needed. We performed unique molecular identifier (UMI) target sequencing on plasma samples and peripheral blood mononuclear cells (PBMCs) from 85 hepatocellular carcinoma (HCC) patients receiving surgical resection, which were divided into an exploration dataset (20 patients) or an evaluation dataset (65 patients). Plasma mutations were identified in pre‐operative plasma, and the mutation variant frequency change (MVFC) between post‐ and pre‐operative plasma was then calculated. In the exploration dataset, we observed that plasma mutations with MVFC < 0.2 were enriched for tumor mutations identified in tumor tissues and had frequency changes that correlated with tumor burden; these plasma mutations were therefore defined as MVFC‐identified tumor mutations. The presence of MVFC‐identified tumor mutations after surgery was related to shorter relapse‐free survival (RFS) in both datasets and thus indicated minimum residual disease (MRD). The combination of MVFC‐identified tumor mutations and Alpha Fetoprotein (AFP) could further improve MRD detection (*P* < 0.0001). Identification of tumor mutations based on MVFC was also confirmed to be applicable with a different gene panel. Overall, we proposed a novel strategy for non‐invasive tumor mutation screening using solely plasma that could be utilized in HCC tumor‐burden monitoring and MRD detection.

## Graphical abstract

Utilization of cfDNA mutation variant frequency change during surgery provides a novel strategy to non‐invasively identify tumor mutations, which is highly compatible with different sequencing panels. The identified tumor mutations show good consistency with tumor burden and enable the accurate detection of minimum residual disease for evaluating patient relapse‐free survival.

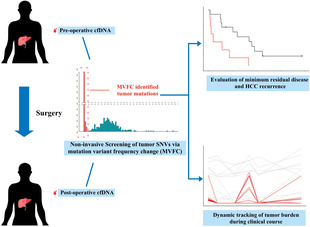

**Abbreviations**
AFP: alpha fetoprotein
AUC: area under the ROC curve
BCLC: Barcelona‐clinic liver cancer
cfDNA: cell‐free DNA
ctDNA: circulating tumor DNA
DCP: Des‐gamma‐carboxy prothrombin
HCC: hepatocellular carcinoma HCC
iDES: integrated digital error suppression
MRD: minimum residual disease
MVFC: mutation variant frequency change
PBMCs: peripheral blood mononuclear cells
RFS: relapse‐free survival
ROC: receiver operating characteristic
SNV: single nucleotide variant
TACE: transarterial chemoembolization
UMI: unique molecular identifier
VAF: variant allele frequency

## 1 Introduction

Hepatocellular Carcinoma (HCC) patients suffer from a high risk of tumor recurrence after surgical resection [1]. Minimal Residual Disease (MRD), defined as the small fraction of tumor cells remaining in human organs after cancer treatments, is a major contributor to HCC metastasis and recurrence [2, 3, 4]. Thus, post‐operative detection of MRD is valuable in accessing HCC recurrent risk [1]. However, due to the nature of MRD, its accurate detection remains challenging [5, 6].

Liquid biopsy based on cell‐free DNA (cfDNA) has provided an opportunity for non‐invasive detection of patients' disease status [7, 8]. In tumor patients, cfDNA contains circulating tumor DNA (ctDNA) originating from tumor tissues, which could be utilized to infer tumor molecular features and thus represents a real‐time surrogate for tumor burden [3, 9]. Tracking tumor mutations (single nucleotide variants, SNVs) via ctDNA has been proved as an effective strategy for monitoring tumor progression, and the development of ultra‐deep sequencing has enabled an accurate determination even for minimal tumor burden [10, 11, 12]. However, one major limitation in utilizing ctDNA is the requirement of prior knowledge for tumor genomic features, which are usually acquired from tissue sampling to avoid interference of DNA fragments originating from other sources [10, 13]. The dependence on tissue sampling not only greatly reduces the non‐invasive value of ctDNA but also presents a major obstacle since tissue samples are sometimes difficult to acquire. Meanwhile, tissue sampling in solid tumors such as HCC is inevitably affected by tumor heterogeneity [14, 15], creating bias in the interpretation of liquid biopsy results. Recent studies have dedicated their efforts to the development of tumor mutation screening strategies without dependence on tissue sampling based on characteristics of mutations originating from different sources (i.e. tumor, hematopoietic clone cell, normal tissue). While numerous sequencing panels have been proposed by researchers to capture tumor mutations [2, 11, 16], their design still faced a dilemma: increasing the target regions greatly increased the false positive rate, while focusing on a small set of highly mutated regions significantly reduced the number of detected tumor mutations. Therefore, a novel strategy capable of non‐invasively discriminating tumor mutations in plasma that is compatible with different panels is valuable and will benefit the accurate detection of MRD [3, 5].

Based on the previous studies reporting that circulating levels of tumor mutations showed dynamic change in consistent with patients' clinical course [17, 18, 19], we further proposed a novel approach to discriminate tumor mutations utilizing the dynamic change of mutation variant frequency during certain clinical events such as surgical operations, which provided a novel direction for tumor mutation identification by integrating mutations' dynamic change. This approach was investigated and evaluated using Unique Molecular Identifier (UMI) sequencing [20] data of a total of 85 HCC patients, which were divided into an exploration dataset and an evaluation dataset.

## 2 Materials and methods

### 2.1 Patients and sample collection

Detailed information regarding patient selection in this article is available in Fig. S1. A total of 85 HCC patients receiving surgical resection at Mengchao Hepatobiliary Hospital of Fujian Medical University were included. Pre‐operative and post‐operative plasma samples were collected for all patients, while additional tumor tissues were collected from 20 patients to validate mutation screening results. The median follow‐up period for enrolled patients was 443 days (range: 29–1869). The sample collection was conducted from August 2015 to August 2019. All human sample usage were in accordance with the principles of the Declaration of Helsinki and the study was approved by the Institution Review Board of Mengchao Hepatobiliary Hospital of Fujian Medical University with approval ID 2020_098_01. Informed written consents were received from all participating patients.

### 2.2 UMI target sequencing and sequencing data analysis

DNA extracted from plasma, tumor tissues and PBMC was subjected to UMI target sequencing based on a customized panel constituted of 549 genes. After quality control, the sequencing reads were aligned to reference human genome (hg19) using consensuscruncher [21]. Then, integrated digital error suppression (iDES) algorithm [22] was deployed to extract nucleic acid base distribution and remove stereotypical errors from technical artifacts. After that, somatic mutations were called with following criteria: global read depth ≥ 100; mutations were supported by double‐strand reads or ≥ 3 single‐strand reads; Variant allele frequency (VAF) is higher than −ln(0.01) divided by global read depth. Mutations that were also detected in corresponding PBMCs (double strands reads or ≥ 3 single‐strand reads and VAF is higher than −ln(0.01) divided by global read depth in PBMCs) were removed to avoid interference. For tumor tissues, only mutations with VAF exceeding 10% were retained as tumor mutations. For plasma, the VAF threshold was set to 0.5% for pre‐operative plasma to screen plasma mutations considering the relatively low VAF.

### 2.3 Screening of plasma mutations

We used mutated allele frequency change between post‐ and pre‐operative plasma, namely Mutation Variant frequency change (MVFC) to evaluate SNVs' capability for reflecting tumor burden. The detailed calculation of MVFC is shown as follows:

$$\text{MVFC}=\frac{{\mathrm{Vaf}}_{\text{post}‐\text{operative}}}{{\mathrm{Vaf}}_{\text{preoperative}}}$$

SNVs with MVFC < 0.2 were considered to show a consistent trend with tumor burden during surgeries. Thus, plasma mutations with MVFC < 0.2 were defined as MVFC‐identified tumor mutations.

### 2.4 Measurement of protein biomarkers in serum

Commercial Lumipulse® G AFP‐N (Alpha‐Fetoprotein; Fujirebio, Tokyo, Japan), and Lumlpulse® G PIVKA‐II Kit (Fujirebio) were used to determine the concentration of Alpha Fetoprotein (AFP) and Des‐gamma‐carboxy prothrombin (DCP) in patients' plasma. The measurement was conducted according to the corresponding manufacturer's instructions.

### 2.5 Determination of MRD

Patients with any MVFC‐identified tumor mutations detected in post‐operative plasma (VAF > 0.1%) were determined as MRD positive based on ctDNA. For AFP and DCP, the cut‐off levels of minimum residual disease indication were set to 20 ng·mL^−1^ and 40 mAU·mL^−1^ respectively according to previous studies [23, 24]. Relapse‐free survival was used as the surrogate endpoint for MRD.

### 2.6 Statistical analysis

All survival analyses comparing RFS of different patient groups were conducted using the Kaplan–Meier method with log‐rank tests [25]. Univariate and multivariate analyses were performed using Cox regression with Cox proportional hazards model. All statistical analysis was conducted on r (version 4.1.0, R Foundation for Statistical Computing, Vienna, Austria). *P* < 0.05 was considered statistically significant.

## 3 Results

### 3.1 Study design

**Fig. 1:**
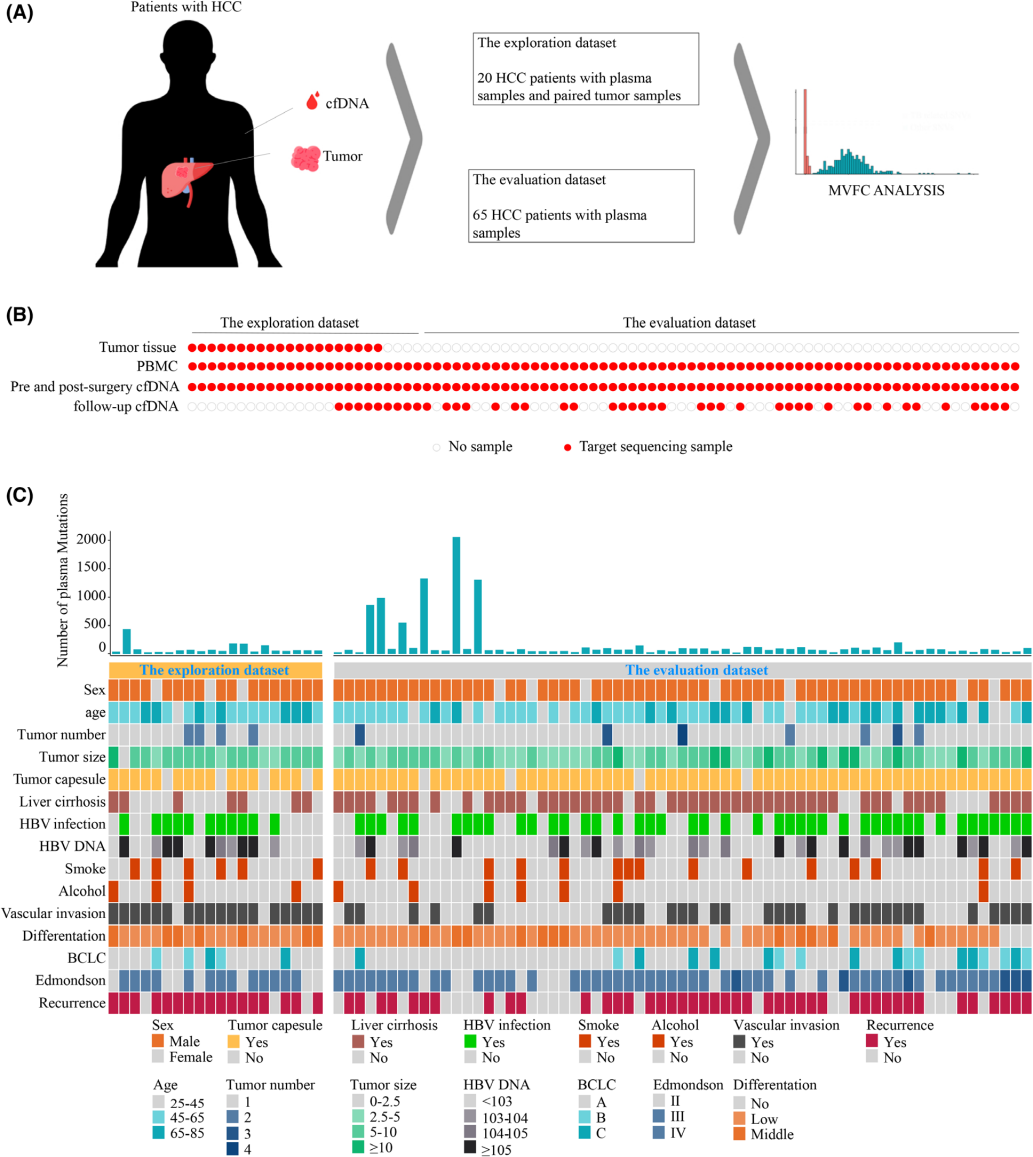
Study design and clinicopathologic information of enrolled HCC patients. (A) A flow chart depicting the detailed design of this study. (B) Information of sample collection and sequencing for enrolled HCC patients. (C) Clinicopathologic information of all enrolled HCC patients. The number of SNVs detected in pre‐operative plasma samples of each patient was shown in the histogram above. Detailed clinical characteristics were shown in the heatmap below. BCLC, Barcelona‐Clinic Liver Cancer; cfDNA, cell‐free DNA; HCC, Hepatocellular Carcinoma; PBMC, peripheral blood mononuclear cell.

A total of 85 HCC patients were enrolled and divided into two datasets, namely an exploration dataset and an evaluation dataset. An illustration of patient selection was provided in Fig. S1. The detailed study design was shown in Fig. 1A. For the exploration dataset containing 20 HCC patients, plasma samples before and after surgical operations as well as at available follow‐up timepoints, PBMCs and tumors were collected for UMI target sequencing using an in‐house customized panel. For the evaluation dataset containing 65 HCC patients, only plasma samples before and after surgical operations and corresponding PBMCs were collected and sequenced. Plasma mutations were identified in pre‐operative plasma, and available tumor tissues were sequenced to identify tumor mutations. The detailed information for sample sequencing was shown in Fig. 1B, and the clinical information of enrolled patients was illustrated in Fig. 1C. For plasma, tumor and PBMC samples, the average sequencing depth is 1558.16×, 1377.83× and 847.42× respectively. The statistics of sequencing coverage and quality for all included samples was available in Table S1 and the detailed clinical information for enrolled patients was provided in Table S2.

### 3.2 Dynamic change of variant frequency could discriminate tumor mutations in plasma

**Fig. 2:**
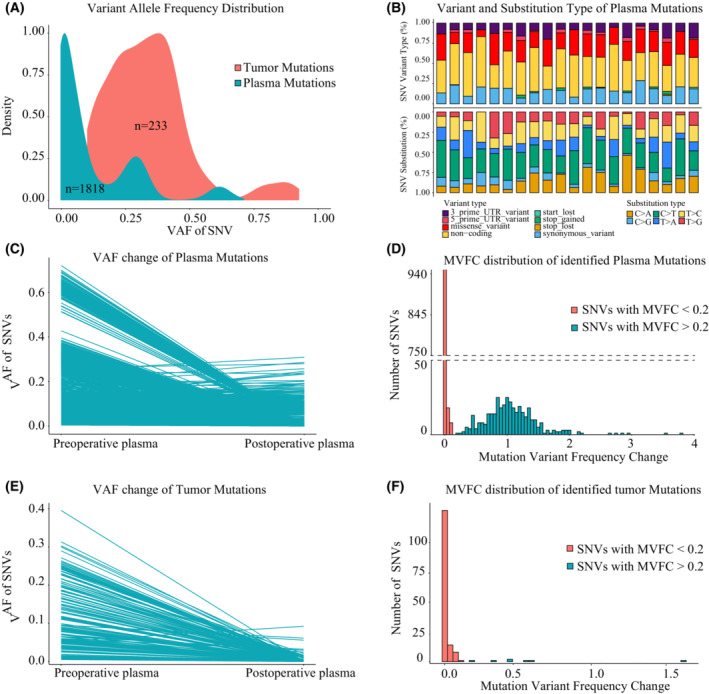
The Mutation Variant Frequency Change (MVFC) patterns of plasma and tumor mutations during surgical operations in the exploration dataset. (A) Variant allele frequency distribution of detected plasma mutations in pre‐operative plasma and tumor mutations identified from tumor tissues. (B) Variant types and SNV substitution distribution of detected plasma mutations. (C) The variant frequency change of plasma mutations between pre‐ and post‐operative plasma samples. (D) The distributions of Mutation Variant Frequency Change (MVFC) of plasma mutations. Plasma mutations were categorized into two types according to MVFC (threshold 0.2) and colored differently. (E) The variant frequency change of tumor mutations identified from tumor tissue between pre‐ and post‐operative plasma samples. (F) The distributions of Mutation Variant Frequency Change (MVFC) of tumor mutations identified from tumor tissue. Tumor mutations were categorized into two types according to MVFC (threshold 0.2) and colored differently. MVFC, Mutation Variant Frequency Change; SNV, single nucleotide variant; VAF, Variant allele frequency.

As pointed out by previous reports, the VAF of tumor mutations in plasma should dynamically change according to tumor burden. Therefore, we hypothesized that the VAF change in plasma during clinical events might discriminate tumor mutations. To prove this, we identified plasma mutations in pre‐operative plasma for the exploration dataset. As we expected, a large number of mutations were detected in pre‐surgery plasma, with an average of 90.9 (range: 24–578) for each patient. The distributions of VAF and functional categorization of identified plasma mutations were illustrated in Fig. 2A,B. Then, we compared their VAF between pre‐ and post‐surgery plasma. As shown in Fig. 2C, plasma mutations showed different VAF change trends. To better illustrate the VAF change, we introduced a parameter named Variant frequency fold change (MVFC), which was defined as the ratio between post‐operative VAF and pre‐operative VAF.

Mutation variant frequency change distribution showed that plasma mutations could be categorized into two classes (Fig. 2D): mutations with VAF decrease after surgery (MVFC < 0.2, red) and mutations with VAF unaffected by surgery (MVFC > 0.2, blue).

Since surgery greatly reduced tumor burden, we deduced that plasma mutations with MVFC < 0.2 represented mutations originating from tumor tissues. To validate this, we further identified tumor mutations in corresponding tumor tissues (mutation calling process of tumor mutations was available in the Section 2) and determined their MVFC. As illustrated in Fig. S2, a total of 233 tumor mutations (average:11.65, range: 2–26) were identified in tumor and a majority of them (157/233) were also detected in pre‐operative plasma. The gene distribution of identified tumor mutations was shown in Fig. S3. Comparison of VAF of these tumor mutations between pre‐ and post‐surgery plasma showed that most of them demonstrated dramatic decrease after surgery (Fig. 2E). MVFC analysis further revealed that these mutations showed MVFC < 0.2 (Fig. 2F).

**Fig. 3:**
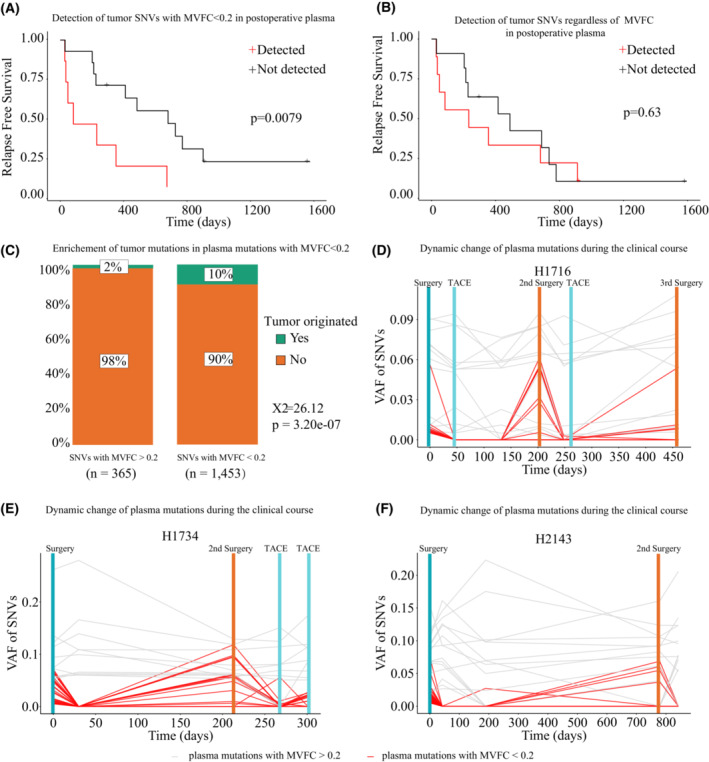
Screening plasma mutations based on MVFC could enrich mutations reflecting patients' tumor burden in the exploration dataset. (A) The Kaplan–Meier analysis (log rank test) showed that detectable levels of tumor mutations with MVFC < 0.2 in post‐operative plasma were significantly associated with tumor recurrence. (B) The Kaplan–Meier analysis (log rank test) showed that detectable levels of all tumor mutations without the discrimination of MVFC in post‐operative plasma were not associated with tumor recurrence. (C) Chi‐square test confirmed that plasma mutations with MVFC < 0.2 could significantly enrich tumor mutations identified in tumor tissues (X^2^ = 29.25, *P* = 6.38e‐08). (D) The variant frequency of plasma mutations with MVFC < 0.2 was highly consistent with tumor burden during clinical courses in patient H1716. (E) The variant frequency of plasma mutations with MVFC < 0.2 was highly consistent with tumor burden during clinical courses in patient H1734. (F) The variant frequency of plasma mutations with MVFC < 0.2 was highly consistent with tumor burden during clinical courses in patient H2143. MVFC, Mutation Variant Frequency Change; SNV, single nucleotide variant; TACE, Transarterial chemoembolization.

However, several tumor mutations seemed unaffected by surgery. One possible explanation was that these mutations were the product of sequencing noise or interference from other cfDNA origins. Since post‐operative detection of tumor mutation in plasma should reflect the tumor residues after surgery, it should also be related to early recurrence. Therefore, Kaplan–Meier analysis was deployed and the results showed that post‐operative detection of tumor mutations with MVFC < 0.2 in plasma was significantly associated with shorter RFS (Fig. 3A, *P* = 0.0079), supporting that this fraction of tumor mutations was related to early recurrence, while tumor mutations with MVFC > 0.2 failed to evaluate patients' RFS, proving that they might be induced by sequencing noise (Fig. 3B, *P* = 0.63).

The above observations confirmed that tumor mutations had distinct characteristics in MVFC distribution, and therefore could be discriminated based on MVFC. Indeed, Chi‐square tests showed tumor mutations were significantly enriched in plasma mutations with MVFC < 0.2 (*P* < 0.0001, Fig. 3C). Using three patients with available sequential follow‐up plasma samples, we further traced the VAF of plasma mutations during patients' clinical courses. The results confirmed that plasma mutations with MVFC < 0.2 presented real‐time surrogates for tumor burden, while plasma mutations with MVFC > 0.2 did not (Fig. 3D–F). Therefore, we could conclude that MVFC could indeed discriminate tumor mutations in plasma. For convenience, we would refer to plasma mutations with MVFC < 0.2 as MVFC‐identified tumor mutations in subsequent parts of this article.

### 3.3 Tumor mutations identified by MVFC could indicate the presence of MRD

**Fig. 4:**
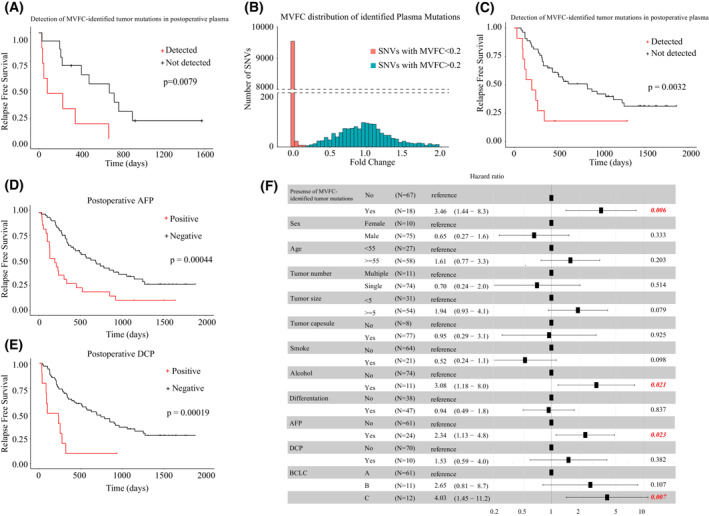
MVFC‐identified tumor mutations could indicate MRD. (A) The Kaplan–Meier analysis (log rank test) showed that the post‐operative levels of MVFC‐identified tumor mutations were significantly associated with tumor recurrence in the exploration dataset. (B) The distributions of Mutation Variant Frequency Change (MVFC) of plasma mutations in the evaluation dataset. Plasma mutations were categorized into two types according to MVFC (threshold 0.2) and colored differently. (C) The Kaplan–Meier analysis (log rank test) showed that detectable levels of MVFC‐identified tumor mutations in post‐operative plasma were significantly associated with tumor recurrence in the evaluation dataset. (D) The Kaplan–Meier analysis (log rank test) showed that positive level of AFP in post‐operative plasma was significantly associated with tumor recurrence. (E) The Kaplan–Meier analysis (log rank test) showed that positive level of DCP in post‐operative plasma was significantly associated with tumor recurrence. (F) Multi‐variate Cox analysis of clinical parameters and presence of MVFC‐identified tumor mutations on RFS. Red represents significance. AFP, Alpha Fetoprotein; BCLC, Barcelona‐Clinic Liver Cancer; DCP, Des‐gamma‐carboxy prothrombin; MVFC, Mutation Variant Frequency Change.

As described above, post‐operative presence of tumor mutations in plasma reflected detectable MRD after surgery. Therefore, we further explored the clinical application of MVFC‐based mutation screening strategy by investigating its capability to non‐invasively detect MRD. Not surprisingly, division of HCC patients based on post‐operative presence of MVFC‐identified tumor mutations in plasma achieved the same result as post‐operative presence of tumor mutations, and therefore MVFC‐identified tumor mutations are well correlated with patients' prognosis (Fig. 4A, *P* = 0.0079).

Additional Receiver operating characteristic (ROC) analysis confirmed that post‐operative detection of MVFC‐identified tumor mutations in plasma could discriminate patients with or without early recurrence (less than 6 months after surgery) with an Area under the ROC Curve (AUC) of 0.8 (Fig. S4). Considering that MVFC‐identified tumor mutations presented real‐time surrogates for tumor burden, this observation proved that the presence of MVFC‐identified tumor mutations in post‐operative plasma could indicate MRD.

To evaluate this tumor mutation screening method in a larger dataset with the same sequencing strategy, we applied MVFC based screening approach in the evaluation dataset. Analysis showed that MVFC distribution of all plasma mutations identified by UMI sequencing in the evaluation dataset was very similar to that in the exploration dataset, suggesting that our division of enrolled patients did not produce noticeable bias (Fig. 4B). After dividing plasma mutations based on their MVFC, we found that post‐operative presence of MVFC‐identified tumor mutations in plasma could discriminate patients with/without early HCC recurrence, supporting that their detection indicated MRD (Fig. 4C, *P* = 0.0032), and patients with these mutations after surgery showed significantly worse prognosis. These results proved the value of MVFC‐based mutation screening strategy in non‐invasive MRD detection. After evaluation, two datasets were combined for downstream analysis.

### 3.4 Combining MVFC‐identified tumor mutations with AFP provided a more accurate evaluation of patients' prognosis

**Fig. 5:**
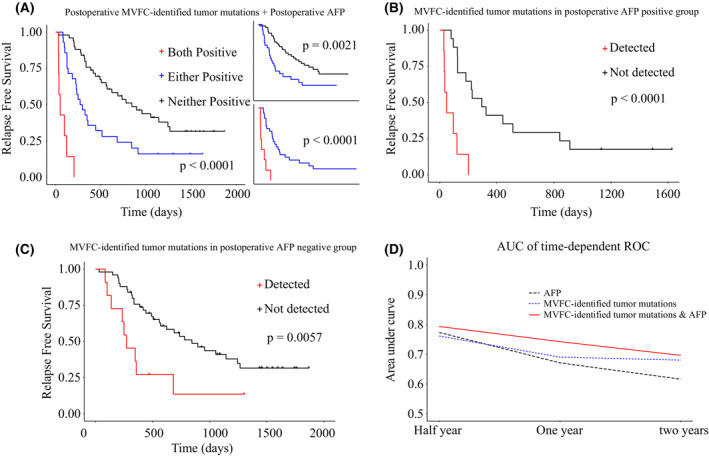
Combining MVFC‐identified tumor mutations and AFP achieved better evaluation of MRD. (A) The Kaplan–Meier analysis (log rank test) of post‐operative presence of MVFC‐identified mutations combined with post‐operative positive AFP on RFS. (B) The Kaplan–Meier analysis (log rank test) of post‐operative presence of MVFC‐identified mutations in patients with positive levels of post‐operative AFP. (C) The Kaplan–Meier analysis (log rank test) of post‐operative presence of MVFC‐identified mutations in patients with negative levels of post‐operative AFP. (D) Comparison of the performance in indicating patients' RFS of three strategies (AFP, MVFC‐identified tumor mutations, combination of both) at different time periods evaluated by the estimated area under the time dependent ROC curve. AFP, Alpha Fetoprotein; MVFC, Mutation Variant Frequency Change.

To compare the prognostic performance of the MVFC‐identified tumor mutations and known biomarkers including post‐operative AFP and DCP [4, 23], we first used univariate cox analysis to screen clinical features and biomarkers with prognostic value. Identified clinical features related to RFS included age, tumor number, tumor size, tumor differentiation and smoking/alcohol assumption history, and AFP and DCP were both significantly related to RFS (Fig. 4D,E). Multi‐variate cox analysis further revealed that post‐operative detection of MVFC‐identified tumor mutations, alcohol, AFP and BCLC were independent risk factors of RFS (Fig. 4F). Therefore, MVFC‐identified tumor mutations and AFP were combined to provide an integrated evaluation of patients' prognosis. As shown in Fig. 5A, combining these two biomarkers greatly increased the performance of prognostic evaluation. Furthermore, MVFC‐based mutation detection could implement AFP in early HCC recurrence, showing prognostic values in both post‐operative AFP positive and negative patients (Fig. 5B,C).

To further determine the prognostic value of MVFC‐based mutation detection for different time periods, time‐dependent ROC curves were employed. Patients classified by the joint markers were considered positive if at least one biomarker was positive. As shown in Fig. 5D, MVFC‐identified tumor mutations combined with AFP showed the best prognostic efficiency than either of the two biomarkers. Meanwhile, each strategy showed the highest performance for discriminating patients with high risk of HCC recurrence at half year compared to both 1‐ and 2‐year time points, which is within expectation since half a year is an experimental time threshold for MRD detection.

### 3.5 Validation of MVFC distribution patterns in another gene panel

**Fig. 6:**
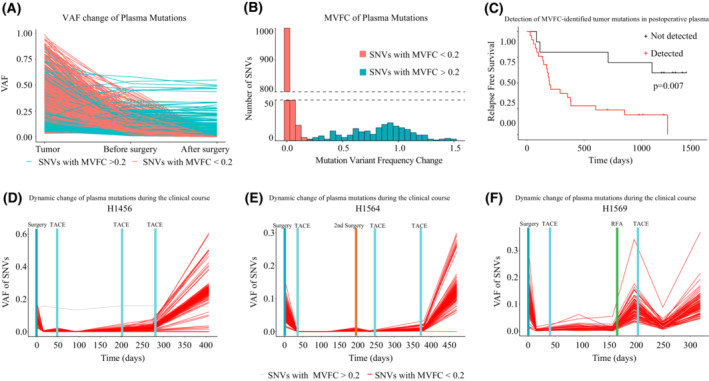
The MVFC distribution pattern of plasma mutations in another HCC cohort. (A) Similar variant frequency changes of plasma mutations were observed in pre‐ and post‐operative plasma samples. (B) The distributions of Mutation Variant Frequency Change (MVFC) of plasma mutations. Plasma mutations were categorized into two types according to MVFC (threshold 0.2) and colored differently. (C) The Kaplan–Meier analysis (log rank test) showed that presence of MVFC‐identified tumor mutations in post‐operative plasma was significantly associated with tumor recurrence. (D) The variant frequency of MVFC‐identified tumor mutations was highly consistent with tumor burden during clinical courses in patient H1456. (E) The variant frequency of MVFC‐identified tumor mutations was highly consistent with tumor burden during clinical courses in patient H1564. (F) The variant frequency of MVFC‐identified tumor mutations was highly consistent with tumor burden during clinical courses in patient H1569. MVFC, Mutation Variant Frequency Change; SNV, single nucleotide variant; TACE, Transarterial chemoembolization; VAF, Variant allele frequency.

To assess whether MVFC‐based tumor mutation identification is compatible with other gene panels, we analysed another HCC cohort with a different customized designed panel used in our published research [18]. This study included 35 patients, with the designed gene panel specifically capturing mutations detected in tumor tissues. Thus, all plasma mutations identified in this cohort were also identified in tumor. Consistently, these SNVs showed VAF change following two different patterns (Fig. 6A) and the MVFC distribution was similar to the results presented above (Fig. 6B). Kaplan–Meier analysis confirmed that patients with detectable MVFC‐identified tumor mutations after surgery had significantly shorter RFS (Fig. 6C, *P* = 0.007), which indicated that the post‐operative detection of these mutations reflected the presence of MRD. Monitoring MVFC‐identified tumor mutations in follow‐up plasma samples, we observed that their VAF was highly consistent with tumor burden (Fig. 6D–F). These results showed that the MVFC‐based mutation screening strategy is not restricted to gene panels, and thus could be widely applied in sequencing data using numerous gene panels with promising clinical application potential. In summary, our study proposed a novel non‐invasive strategy for tumor mutation identification that is highly compatible with gene panels designed for various scenarios.

## 4 Discussion

Liquid biopsy has become an important non‐invasive replacement of traditional tissue biopsy to acquire tumor information with much less risk of complication [8, 10]. Tracking tumor mutations via ctDNA was considered as one of the most promising directions for dynamical and accurate evaluation of tumor burden [2, 7, 11, 18]. However, how to isolate tumor mutations from plasma mutations still remained challenging without prior knowledge from tissue sampling [26]. Our study proposed a novel approach by addressing the problem reversely: since tumor mutations can dynamically reflect tumor burden, whether the dynamic change of VAF is accordant to tumor burden could be utilized to identify such mutations. Our analysis showed that MVFC of plasma mutations between post and pre‐operative timepoints followed distinct patterns and formed two mutation clusters: plasma mutations with MVFC < 0.2 (termed as MVFC‐identified tumor mutations) and plasma mutations with MVFC > 0.2. The levels of MVFC‐identified tumor mutations in plasma were highly correlated with patients' tumor burden during the clinical courses and could serve as an excellent post‐operative indicator of MRD, proving the effectiveness of our approach in tumor mutation screening. These results prompted an intriguing new direction for the development of cfDNA‐based liquid biopsy and its application in prognosis evaluation.

Numerous studies have dedicated their efforts to building customized gene panels to focus on tumor hotspot genes, aiming to improve the detection and tracking of tumor mutations [11, 12, 27, 28]. Although our study was mainly conducted on an in‐house gene panel, we also investigated MVFC distribution patterns on the sequencing data using a different customized gene panel [18]. The results showed that MVFC patterns of plasma mutations were not restricted to a specific gene panel. This observation proved that identifying tumor mutations using MVFC was compatible with other gene panels designed with different strategies, which could further improve the application of our approach. However, mutation calling parameters should be adjusted for different gene panels to acquire plasma mutation with high confidence. Meanwhile, the MVFC threshold might also be affected by the gene panels and clinical scenarios, and therefore a MVFC distribution plot should be drawn to determine the dividing point of the two clusters of mutations.

Due to the high recurrence nature of tumor, the management of various cancers including HCC is a lengthy process and liquid biopsy's non‐invasive advantage provided the possibility to monitor tumor burden in real‐time [3, 8]. Noteworthily, screening tumor mutation using MVFC distribution is well compatible with the analysis of time‐series plasma samples since each time‐point provides a chance to further improve the accuracy of extracting tumor mutations from plasma. Meanwhile, since MVFC reflects how tumor cells respond to the clinical treatments, tumor clonal evolution [15, 29, 30] should also affect the distribution of plasma mutations, Therefore, a detailed investigation of MVFC distribution characteristics might be utilized to track tumor clonal structure change. Future studies including long‐term follow‐up samples are needed to fully explore the clinical utility of MVFC.

Although our study provides a novel direction for non‐invasive tumor mutation identification, there are certain shortcomings that require further improvements. First, it should be noted that utilization of MVFC in screening tumor mutations requires knowledge of how certain clinical events affect tumor burden, and its accuracy might be reduced when change in tumor burden is difficult to evaluate. Second, the performance of MVFC based mutation identification depends on the magnitude of tumor burden change, and less significant change might not result in a clear division between tumor mutations and other plasma mutations. Further efforts are needed to improve MVFC based mutation identification in scenarios other than surgery.

In summary, our study provides a novel perspective for non‐invasively identifying tumor mutations to reflect patients' disease status and therapeutic response. This approach is compatible with different gene panels and could be further combined with time series blood samples to improve its clinical utilization.

## 5 Conclusion

Screening cfDNA mutations based on mutation variant frequency change (MVFC) between post‐ and pre‐operative timepoints provided a novel non‐invasive approach for accurately discriminating tumor mutation, which is in good compatibility with different gene panels. Post‐operative presence of identified tumor mutations showed significant correlation with patients' relapse free survival, providing a potent tool for MRD detection, which could be further combined with AFP for more accurate evaluation.

## Conflict of interest

The authors declare no conflict of interest.

## Author contributions

In this study, the contribution of each author is listed as follows: XL, HZ, and GC involved in study concept and design; GC, FP, XiuD, YZ and ZC involved in data collection and organization; ZL, FP, XiaD, JH, JL and YG involved in data processing and analysis; GC, FP, LH and LQ involved in the interpretation of data; XL, GC, JL and HZ reviewed the article; XL and HZ involved in study supervision.

### Peer review

The peer review history for this article is available at https://www.webofscience.com/api/gateway/wos/peer‐review/10.1002/1878‐0261.13498.

## Supporting information


**Fig. S1.** Patient selection.
**Fig. S2.** Number of tumor mutations from paired tumor tissues that were detected in pre‐operative plasma for the exploration dataset.
**Fig. S3.** Gene distribution of identified tumor mutations.
**Fig. S4.** AUC of post‐operative detection of MVFC‐identified tumor mutations in discriminating HCC patients with early recurrence.

**Table S1.** Sequencing coverage and quality statistics.
**Table S2.** Clinical statistics of HCC patients included in analysis.

## Data Availability

Raw data of this study has been uploaded to the Genome Sequence Archive in BIG Data Center, Beijing Institute of Genomics (BIG), Chinese Academy of Sciences with accession number HRA003229 (https://ngdc.cncb.ac.cn/gsa‐human/browse/HRA003229) and will be made available upon reasonable request.
