# Supplementary material for: Identification of tumor mutations in plasma based on mutation variant frequency change (MVFC)
Source: Mol Oncol. 2023 Aug 7;17(9):1871–83. doi: 10.1002/1878-0261.13498 (PMC10483605; doi:10.1002/1878-0261.13498)
Supplement: Supplementary file 1 — Fig. S1. Patient selection. Fig. S2. Number of tumor mutations from paired tumor tissues that were detected in pre‐operative plasma for the exploration dataset. Fig. S3. Gene distribution of identified tumor mutations. Fig. S4. AUC of post‐operative detection of MVFC‐identified tumor mutations in discriminating HCC patients with early recurrence. [file MOL2-17-1871-s002.docx]

# Supplementary material

Table of contents

Supplementary Figure 1. Patient Selection

Supplementary Figure 2. Number of Tumor Mutations from Paired Tumor Tissues That Were Detected in Pre-Operative Plasma for the Exploration Dataset

Supplementary Figure 3. Gene Distribution of Identified Tumor Mutations

Supplementary Figure 4. AUC of Post-Operative Detection Of MVFC-Identified Tumor Mutations in Discriminating HCC Patients with Early Recurrence

Supplementary Figure 1. Patient Selection


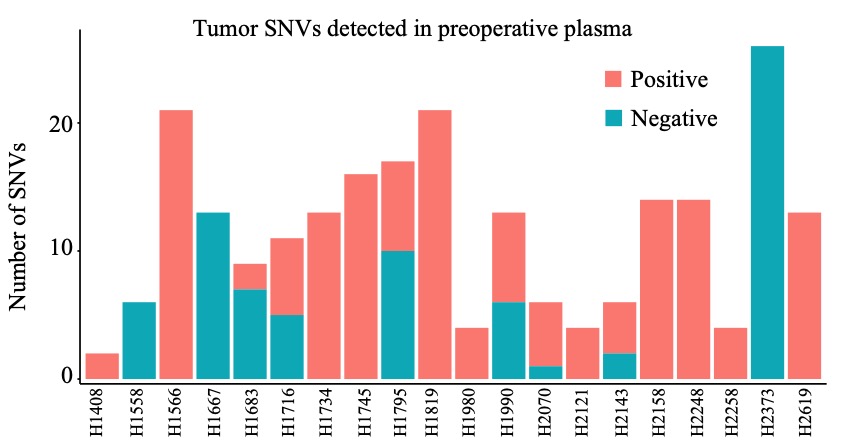


Supplementary Figure 2. Number of Tumor Mutations from Paired Tumor Tissues That Were Detected in Pre-Operative Plasma for the Exploration Dataset

Supplementary Figure 3. Gene Distribution of Identified Tumor Mutations


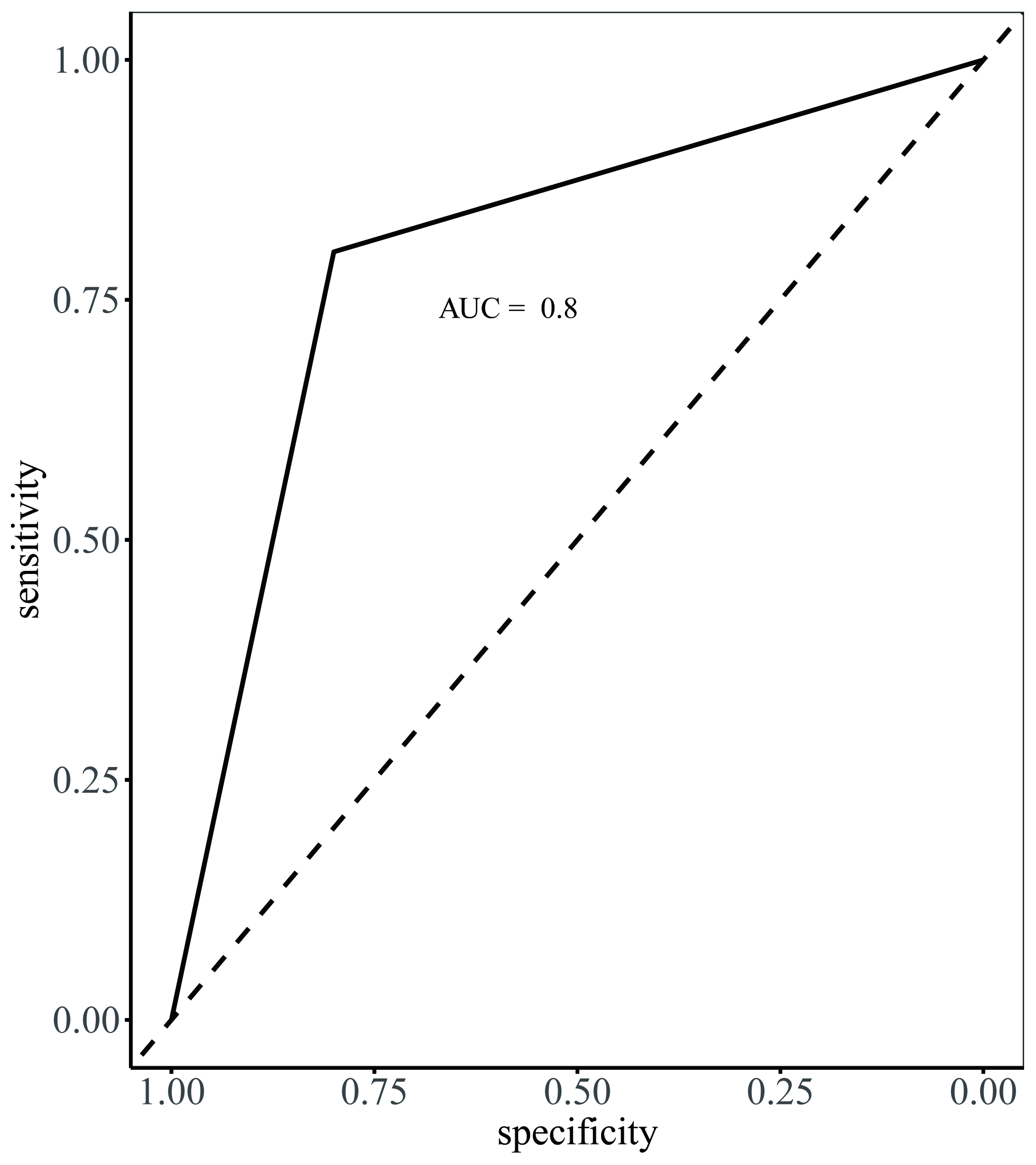


Supplementary Figure 4. AUC of Post-Operative Detection Of MVFC-Identified Tumor Mutations in Discriminating HCC Patients with Early Recurrence
